# Melkersson–Rosenthal syndrome in the context of sarcoidosis: a case report 

**DOI:** 10.1186/s13256-021-03044-5

**Published:** 2021-10-04

**Authors:** J. Casper, S. Mohammad-Khani, J. J. Schmidt, J. T. Kielstein, T. Lenarz, H. Haller, Annette D. Wagner

**Affiliations:** 1 Department of Nephrology and Hypertension, Hanover Medical School, Carl-Neuberg-Strasse 1, 30625 Hanover, Germany; 2 Department of Otolaryngology (ENT), Hanover Medical School, Carl-Neuberg-Strasse 1, 30625 Hanover, Germany; 3 Department of Nephrology, Städtisches Klinikum Braunschweig, Freisestraße 9-10, 38118 Brunswick, Germany

**Keywords:** Case report, Melkersson–Rosenthal syndrome, Clofazimine

## Abstract

**Background:**

Melkersson–Rosenthal syndrome is a rare disease characterized by the triad of recurrent orofacial swelling with facial paralysis and fissured dorsal tongue. Histologically, noncaseating granulomatous inflammation occurs that confirms the diagnosis. Overlaps between granulomatous diseases such as sarcoidosis and Crohn’s disease are described. Systemic corticosteroid therapy is the treatment of choice for acute attacks.

**Case presentation:**

We here present a case of a 59-year-old White woman suffering from Melkersson–Rosenthal syndrome with a past history of sarcoidosis on therapy with leflunomide in combination with low-dose tacrolimus successfully treated with the anti-leprosy drug clofazimine after failure of systemic steroid therapy.

**Conclusions:**

We propose clofazimine as an alternative treatment in steroid-refractory cases.

## Introduction

Melkersson–Rosenthal syndrome (MRS) is a rare syndrome composed of the triad of recurrent orofacial swelling with facial paralysis and fissured dorsal tongue (lingua plicata) [1, 2].

Melkersson (1928) first described a case with facial palsy and orofacial edema caused by noncaseating granulomatous infiltration in the orofacial region [1]. The term MRS was proposed by Rosenthal in 1932 when he reported three cases suffering from fissured tongue in addition to facial paralysis and orofacial edema [2]. Oligosymptomatic and monosymptomatic forms can occur and are more common than the complete triad. Orofacial swelling, especially edema of the lips, is the most common monosymptomatic presentation of MRS (also termed Meischer’s syndrome or cheilitis granulomatosa) [3, 4].

Besides swelling of the lips and lingua plicata, other oral manifestations such as oral mucosal ulceration and gingival overgrowth have also been described. Tissue edema is attributed to lymphatic and vascular disruption by granulomas [2, 3].

The clinical presentation of the mentioned clinical features supported by the histopathologic findings of noncaseating granulomatous inflammation confirms the diagnosis of MRS. The occurrence of granulomatous lesions in the orofacial region may also be a component of Crohn’s disease or sarcoidosis, or a result of tissue reaction to foreign bodies or granulomatous infectious diseases such as tuberculosis. Histopathological and clinical overlaps between diseases caused by noncaseating granulomatous infiltrations such as sarcoidosis and Crohn’s disease are well described [5].

Without therapy, spontaneous remission of MRS is possible but rare. Often, the clinical course is characterized by recurrent episodes, mostly with swelling of the lips [6].

Acute attacks with orofacial swellings have been shown to respond to treatment with systemic or intralesional use of corticosteroids [7, 8]. In the case of relapses, long-term use of steroids is limited due to their known side effects. Several nonsteroidal antiinflammatory agents such as clofazimine, hydroxychloroquine, or sulfasalazine are reported to be alternatives to corticosteroid regimens [9, 10]. Therapy of MRS is difficult, and standard treatment schemes are missing.

This article presents a case of MRS associated with sarcoidosis successfully treated with clofazimine after failure of systemic steroid therapy.

## Case presentation

We saw a 59-year-old White female patient with a history of sarcoidosis at the outpatient department in early September 2012. On first sight, the left side of her face was significantly swollen, and she had pain in the preauricular area. The condition had started a few days earlier with swelling of the lips and progressed to left-sided facial edema and unilateral painful preauricular mass.

C-reactive protein was elevated at 54 mg/l (normal concentration up to 5mg/l). Transaminases, cholestasis and retention parameters, urinalysis, and microbiological findings were unremarkable. At this time, the patient was treated with 1 mg tacrolimus 1-0-0, 20 mg leflunomide 1-0-0, and 7.5 mg prednisolone.

For clarification, an inpatient admission to our tertiary center was arranged.

During the clinical examination, we saw a patient in moderate general condition and obese nutritional status with a body weight of 104.7 kg and a height of 168 cm. The patient did not show temperature elevation. Blood pressure was normal at 120/80 mmHg, and pulse rate was 80 beats/minute. On oral examination, the patient showed tender firm swelling of lips, erythematous and swollen gingiva of the upper and anterior teeth, and fissured tongue. In addition, there was generalized gingivitis, and the left side of the face was also markedly swollen and red. Furthermore, there was left-sided fascial paresis. There was no evidence of mouth sores, and odontogenic infections were ruled out. The eyes did not show any pathology. The heart rhythm was regular, the heart sounds S1/S2 were pure, and there were no heart murmurs. Examination of the lungs was without pathological findings. Pressure pain in the area of the abdomen could not be triggered, there was no organomegaly, and bowel sounds were unremarkable. Peripheral edema was absent on both sides, and no calf pressure pain could be induced. The joints were unremarkable. The neurological examination was regular except the facial palsy.

**Fig. 1:**
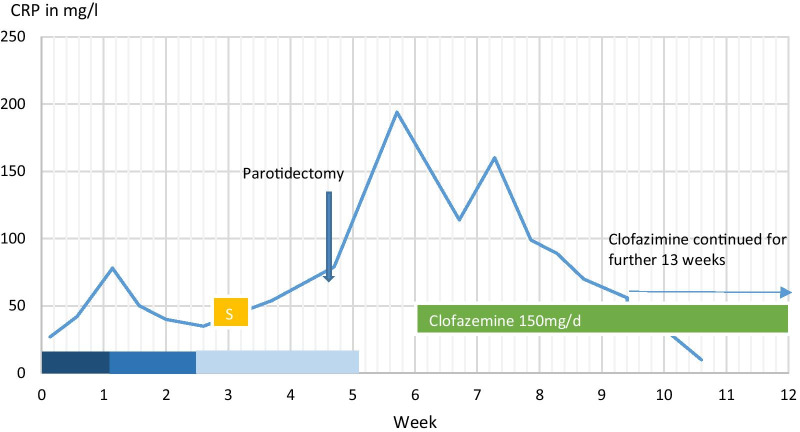
Course of therapy for tissue ulceration and development of C-reactive protein (CRP) levels

Magnetic resonance imaging of the neck on admission showed an enlargement of the left parotid gland with enhancement of contrast medium susceptive of abscess formation. Furthermore, on magnetic resonance imaging, there was a suspicion of a necrotic melting in the left parotid gland, which was displayed with a total extension of approximately 4–5 cm in the three spatial directions. The meltings are displayed with different signal strengths T2-hyperintense and T1-hypointense, and showed both marginal and septal internal contrast enhancement at this time. The findings further included the ramus mandibulae from the dorsal side and extended medially to the pterygoid muscles and laterally to the masseter muscle. The masseter muscle partially showed adjacent contrast enhancement with suspected inflammatory co-reaction. The angle of the jaw showed only small lymph nodes on both sides (Fig. 1).

**Fig. 2:**
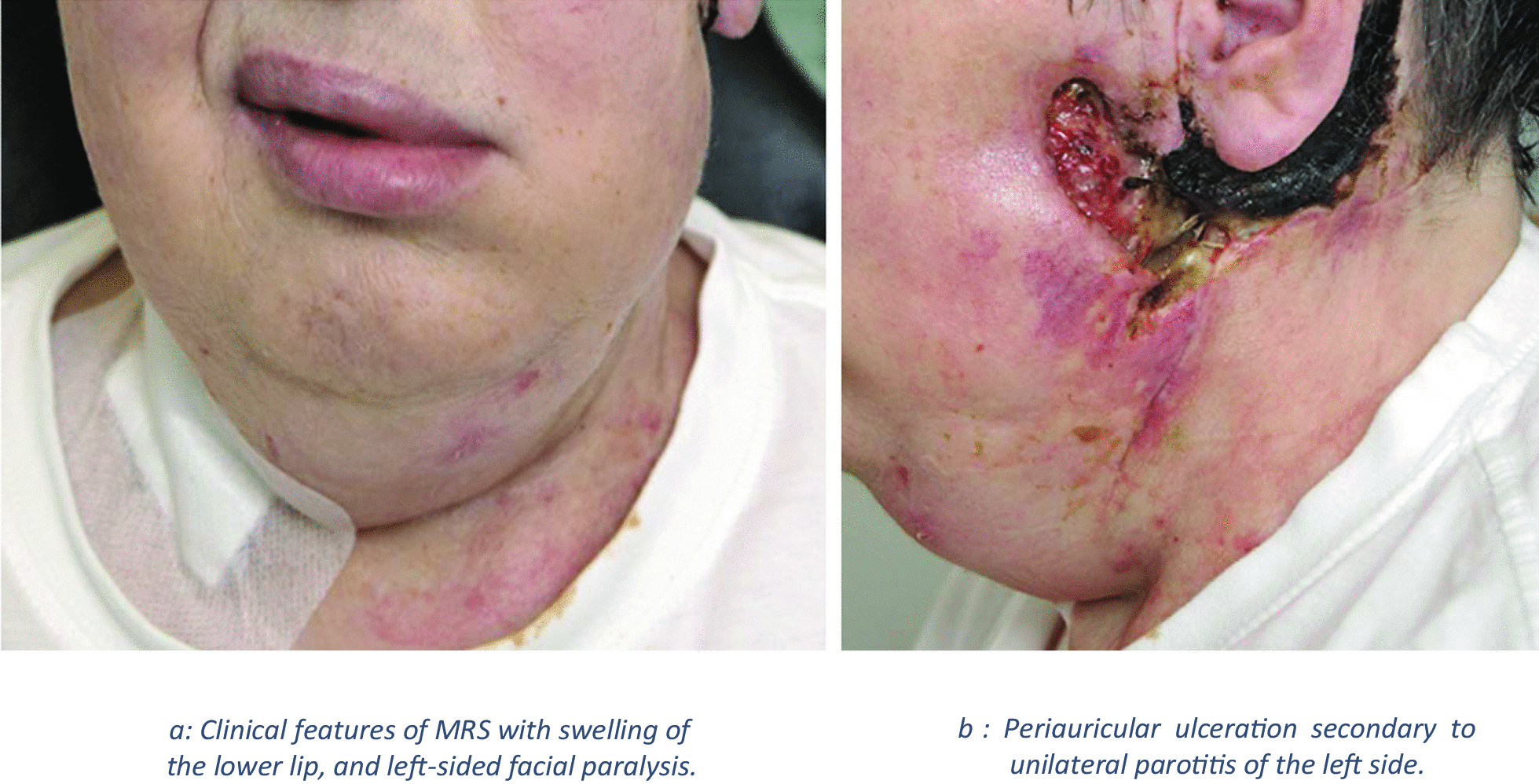
**a** Clinical features of Melkersson–Rosenthal syndrome with swelling of the lower lip, and left-sided facial paralysis. **b** Periauricular ulceration secondary to unilateral parotitis of the left side

After surgical excision of the abscess formation and systemic antibiotic therapy, the patient developed soft-tissue ulceration in pre- and retroauricular area (Fig. 2b). A few days later, a left-sided peripheral facial palsy developed (Fig. 2a). The peripheral left facial palsy was due to swelling and inflammation from parotitis and developed independently of surgery.

No bacteria could be isolated from several wound swabs. Immunoglobulin M (IgM) antibody titer and polymerase chain reaction (PCR) for herpes simplex virus were negative. In addition, a PCR for atypical mycobacteria was done, and again the test was negative. IgM antibody titer and PCR for herpes simplex virus were negative.

Histopathological examinations of the parotid gland revealed necrotizing tissue but also epithelioid granulomas. Malignancy could be excluded.

At that point, a systemic corticosteroid therapy with methylprednisolone 500 mg daily for 3 days was given with no significant improvement of the soft-tissue ulcerations. Therefore, a parotidectomy was performed. Microbiological examinations on bronchoalveolar fluid were negative for pathogenic species. Serological tests such as tuberculosis were also negative. The patient did not report intestinal problems at any time that would have suggested Crohn’s disease. Hence, a colonoscopy did not seem to be justified.

This condition appeared under an immunosuppressive therapy with leflunomide 20 mg once a day in combination with 1 mg tacrolimus daily starting due to pulmonary sarcoidosis, diagnosed 2 years earlier.

### Past medical history

The patient was known to have autoimmune thyroid disease with hypothyroidism prior to the diagnosis of sarcoidosis. In addition, the patient had a history of kidney stone disease and had undergone surgery on the menisci of both knee joints. The patient has an allergy to amoxicillin, and did not have any pregnancies. She has never smoked or had significant alcohol consumption. There was no history of tuberculosis. The patient worked as a commercial employee and was continuously working in the office and not exposed to any noxae.

In October 2009, the patient had a severe feeling of illness with a body weight loss of 15 kg, bloody sputum, and a marked tendency to sweat. Bronchoscopy initially revealed chronic florid inflammation with epitheloid-cell-containing granulomas. Despite slightly elevated antineutrophil cytoplasmic antibody (c-ANCA), there was no evidence of granulomatosis with polyangiitis. Malignancy could also be excluded. From December 2009, the patient was treated with glucocorticoids, and as there was only partial remission in January 2010, the patient was treated with azathioprine for 4 months. Since there was insufficient improvement while taking azathioprine, therapy was switched to methotrexate in May 2010. Methotrexate was then used for a total of 11 months. With disease activity not adequately controlled, methotrexate was discontinued in February 2011, and oral cyclophosphamide was started after four courses of cyclophosphamide pulse therapy. After oral cyclophosphamide therapy, the patient was switched to tacrolimus and leflunomide in September 2011. Recurrent gingivitis occurred from summer 2012 onward. Beside recurrent gingivitis, the patient had orofacial edema and lip swelling as well as a fissured tongue in the past. The final diagnosis based on clinical and histopathological findings was MRS in the context of sarcoidosis.

### Progression under therapy

**Fig. 3:**
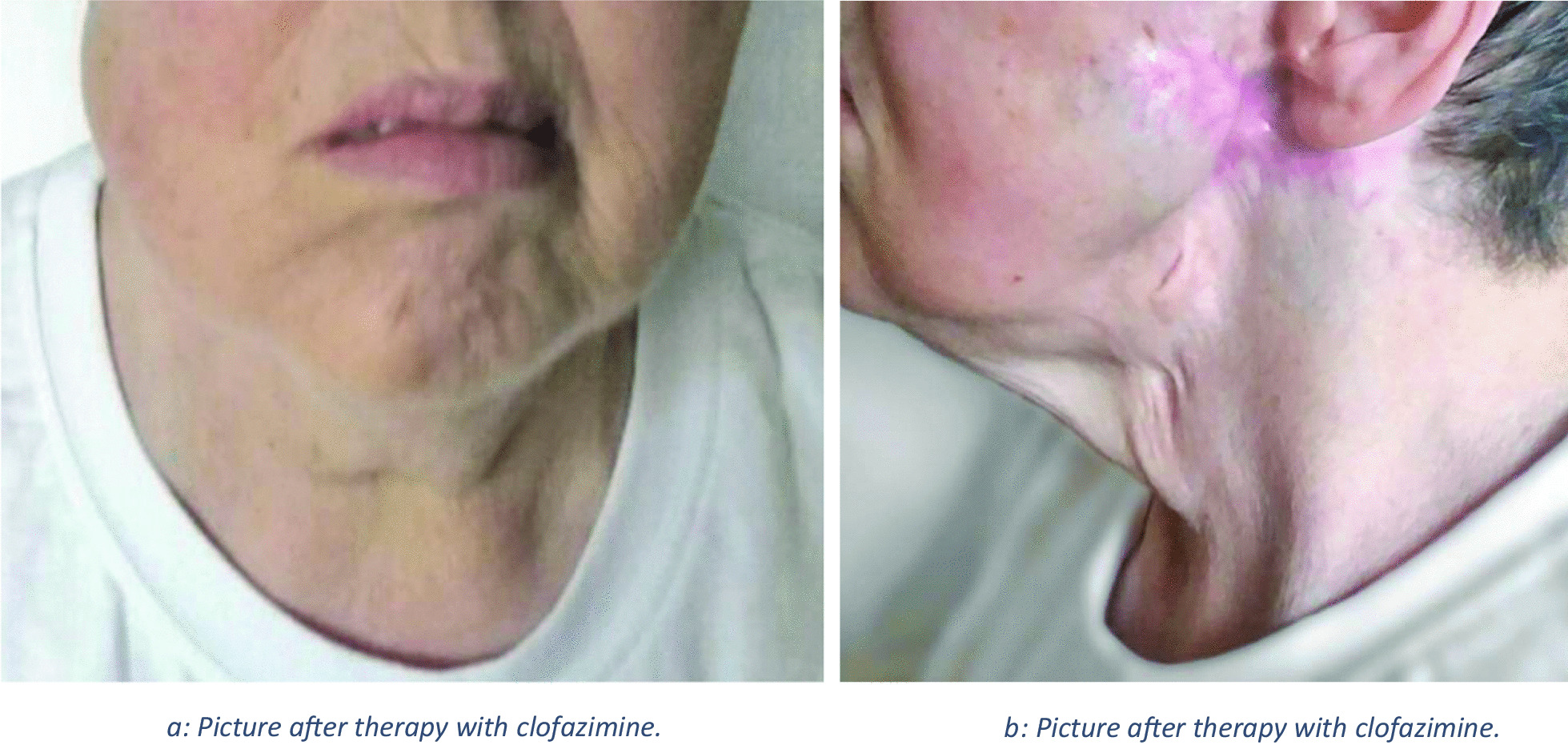
**a** Picture after therapy with clofazimine. **b** Picture after therapy with clofazimine

After treatment with systemic steroids, the labial and facial swelling improved. However, the preauricular ulcerations did not respond to this treatment. At that point of time, the medication with clofazimine 100 mg per day was initiated, which was well tolerated. After 4 weeks of therapy, the tissue ulceration resolved gradually, so that 150 mg of clofazimine three times a week was given for 15 weeks (Fig. 1). After that, clofazimine treatment could be stopped. During 7 years of follow-up, there was no recurrence (Fig. 3a, b).

During the course of the 4-month successful therapy with 150 mg clofazimine 1-0-0, the underlying disease was treated with rituximab. Initially, induction therapy was given with 4 × 375 mg at weekly intervals followed by B-cell and immunoglobulin-controlled therapy. Starting from June 2013, rituximab has been given in combination with 15 mg methotrexate once per week.

## Discussion

MRS is a rare neuromucocutaneus disorder characterized by a triad of recurrent orofacial edema, peripheral facial palsy, and lingua plicata. In 2014, Troiano *et al*. described in a review article that the maxillofacial region and the salivary glands are often involved in the course of sarcoidosis [11]. They further state that oral sarcoidosis usually appears in patients with chronic multisystemic sarcoidosis and often presents as first manifestation [12]. In 1943, Poe *et al*. [13] reported the first documented case of oral sarcoidosis. Since then, there have been more than 60 cases of oral sarcoidosis reported in the literature [14]. In one review of 36 cases, complete triad was present in 9 (25%) patients with MRS [4]. Histologically noncaseating granulomatous inflammation is present.

Based on histomorphological similarity, LIoyd *et al*. suggested that the three conditions might have a common background [15]. However, the origin of this syndrome is still unknown.

Spontaneous remission of MRS is rare. Often, the clinical course is characterized by recurrent episodes, mostly with swelling of the lips [6].

Acute attacks have been shown to respond to treatment with corticosteroids as intralesional injections [8] or as systemic use [7]. Cases with moderate lip swellings can be treated with intralesional triamcinolone 0.1 % injections [8], whereas treatment of patients with more extensive lip or facial swellings require an initial use of systemic steroids. In the case of relapses, long-term use of steroids is limited due to their known side effects. In the literature, several cases of MRS or cheilitis granulomatosa—a monosymptomatic form of MRS—treated successfully with clofazimine have been reported. Sussman *et al*. reported complete remission in 5/10 patients and clinical improvement in 3/10 patients with MRS treated with clofazimine [9].

Tausch and Sonnichsen used clofazimine for therapy in 18 patients suffering from MRS. They reported a decrease in the frequency and intensity of edema in 94% of patients, but persisting improvement throughout a follow-up period of up to 3 years was seen only in 62% of patients [16].

Clofazimine—a prototype riminophenazine antibiotic—has been recommended as a component of the World Health Organization (WHO) triple drug therapy of leprosy since 1962 [17].

*In vitro* studies on the selectivity of clofazimine for Gram-positive bacteria, including mycobacteria, proposed a mechanism of antimicrobial activity caused by disruption of membrane structure and function [18].

In addition to the antimicrobial effect, also antiinflammatory, immunosuppressive activity has been shown when clofazimine was applied in animal model. It has been demonstrated to cause significant suppression of the mitogen- and antigen-driven proliferative responses of isolated T lymphocytes *in vitro* [19, 20].

Side effects of clofazimine are generally mild and dose related [21]. Because of its lipophilicity, clofazimine distributes primarily into fatty tissue, in cells of reticuloendothelial system. Therefore, it enriches in the skin and eyes and causes a reddish-brown discoloration of the skin and conjunctiva. This effect is evident in almost all patients on high doses (300 mg/day) and becomes visible 2–4 weeks after beginning treatment. Avoiding light exposure is recommended to prevent this side effect [22, 23].

Other common side effects such as abdominal pain and transient digestive disturbances are mild to moderate. The adverse effects are gradually reversible after ending therapy [21]. Severe gastrointestinal syndromes such as persistent diarrhea, nausea, weight loss, and enteritis were reported in cases only after use of (> 400 mg) clofazimine daily for long periods in patients suffering from lepra [24, 25]. In patients receiving long-term treatment, elevation of the transaminases, total bilirubin, and blood glucose was seen very rarely [26, 27].

For treatment of MRS and cheilitis granulomatosa, clofazimine is shown to be effective and safe in low dosages (100 mg daily) [9, 16], as is also described in our case.

In humans, the absorption of orally administered drug varies considerably (45–62%) depending on whether the drug is taken with or without food. Without food, the corresponding peak plasma concentration was 30% less. Clofazimine has shown to have a slow elimination, with an elimination half-life about 8–10 days. To avoid the long-lasting accumulation toward the steady state, higher daily loading doses are recommended at the beginning of therapy followed by a daily maintenance dose [28]. Compared with corticosteroids, clofazimine has a better side-effect profile for long-term usage.

## Conclusions

During the course of pulmonary sarcoidosis, the patient developed Melkersson–Rosenthal syndrome that was successfully treated with clofazimine. This description differs from those presented previously in the detailed account of the prehistory and the description of the therapeutic approach.

In summary, it must be emphasized that clofazimine is a possible steroid-sparing therapy for chronic recurrent courses of MRS in patients with a past medical history of sarcoidosis, or may even be a therapeutic option in steroid-refractory patients as described in this case. Current treatment protocols are based on small case studies, and standard treatment schemes for clofazimine usage are missing. Randomized controlled trials are needed to support the drug effectiveness for optimizing the dosing and treatment duration for therapy of MRS with clofazimine.

## Data Availability

Not applicable. All data requests should be submitted to the corresponding author for consideration. Access to anonymized data may be granted following review.
